# Supplementary figures and images for: Perception of color emotions for single colors in red-green defective observers
Source: PeerJ. 2016 Dec 8;4:e2751. doi: 10.7717/peerj.2751 (PMC5149061; doi:10.7717/peerj.2751)

cleanliness

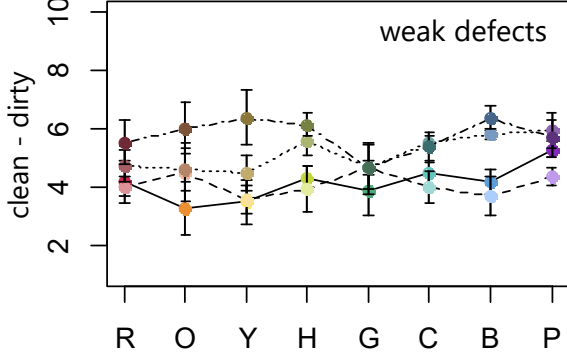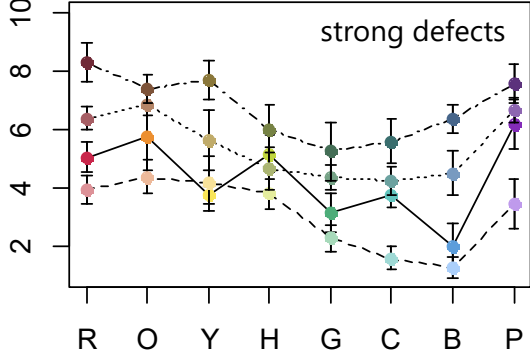

freshness

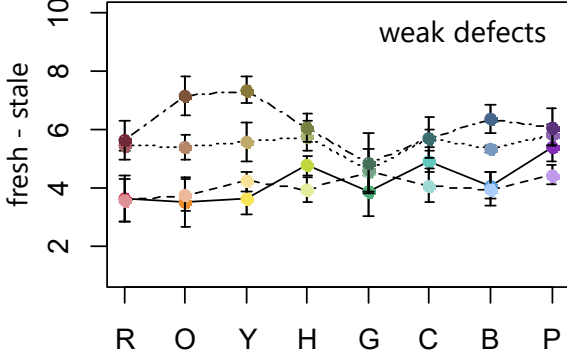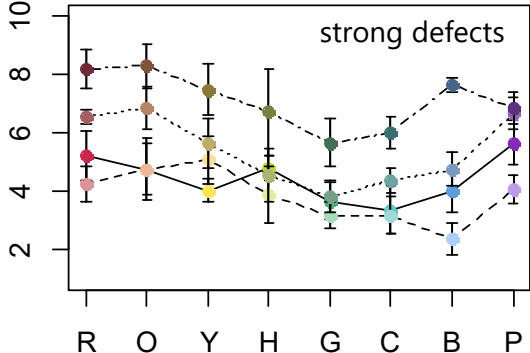

hardness

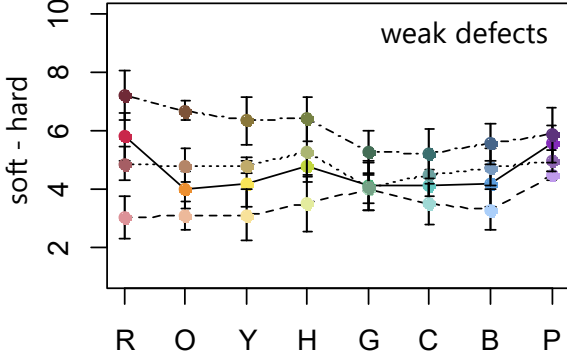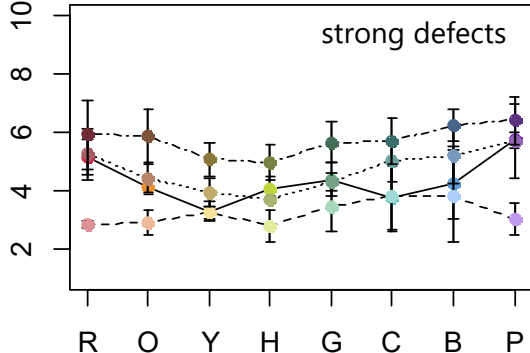

preference

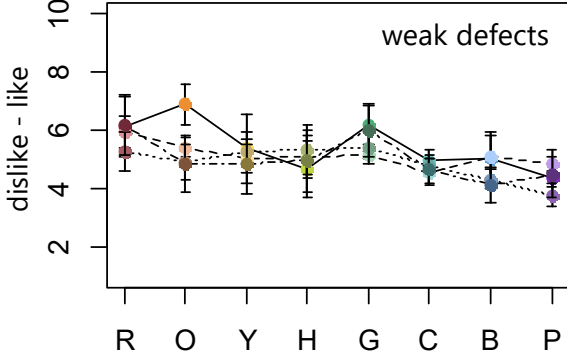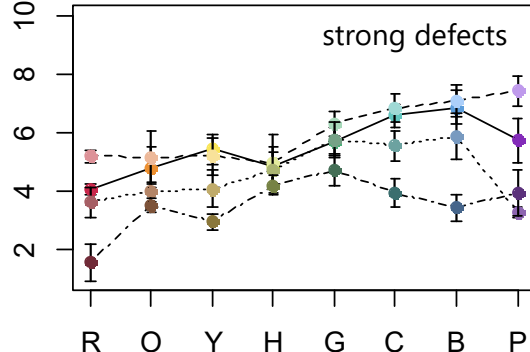

warmth

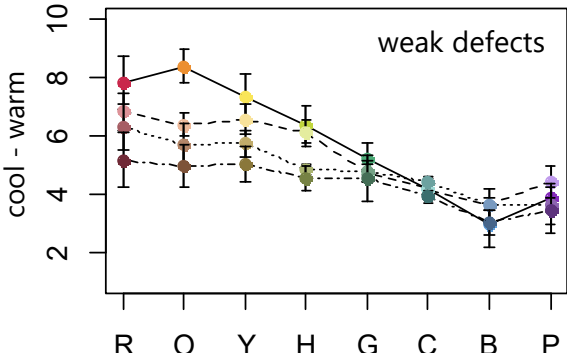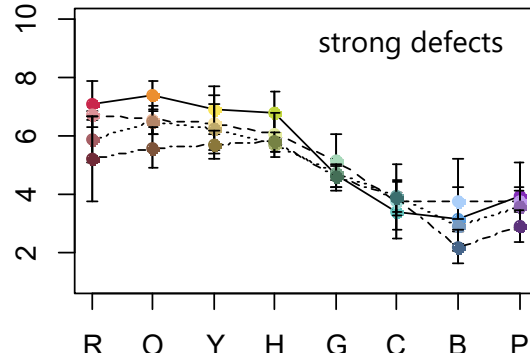

weight

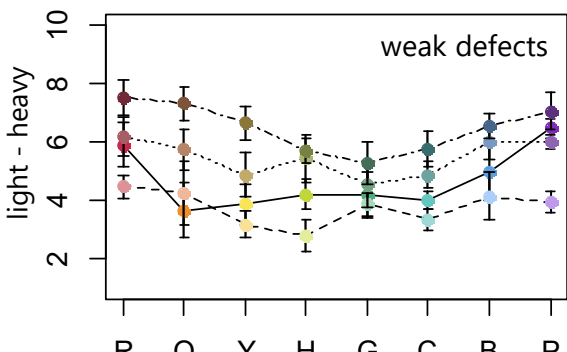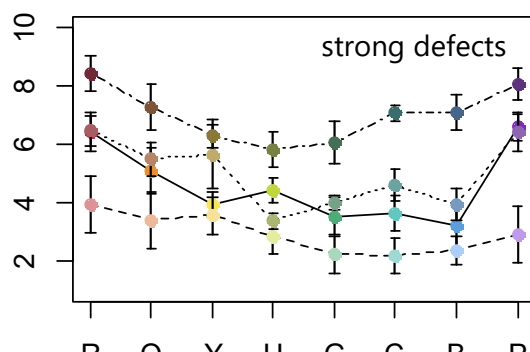

Supplement: Figure S1 — These ratings were averaged for weak red-green defects group (N = 6) and strong defects group (N = 4), separately. [file peerj-04-2751-s001.pdf]
