# Supplementary material for: Perception of color emotions for single colors in red-green defective observers
Source: PeerJ. 2016 Dec 8;4:e2751. doi: 10.7717/peerj.2751 (PMC5149061; doi:10.7717/peerj.2751)
Supplement: Table S1 [file peerj-04-2751-s002.docx]

| color |  | CIE(1931) *x*, *y*, *Y* | | |
| --- | --- | --- | --- | --- |
|  |  | *x* | *y* | *Y* |
| saturated red |  | 0.57 | 0.30 | 19.25 |
| light red |  | 0.41 | 0.32 | 41.43 |
| muted red |  | 0.45 | 0.31 | 19.32 |
| dark red |  | 0.50 | 0.30 | 6.78 |
| saturated orange |  | 0.53 | 0.40 | 41.57 |
| light orange |  | 0.40 | 0.35 | 56.77 |
| muted orange |  | 0.43 | 0.36 | 29.21 |
| dark orange |  | 0.46 | 0.37 | 11.96 |
| saturated yellow |  | 0.43 | 0.46 | 75.43 |
| light yellow |  | 0.22 | 0.51 | 75.50 |
| muted yellow |  | 0.40 | 0.41 | 41.78 |
| dark yellow |  | 0.43 | 0.43 | 19.45 |
| saturated chartreuse |  | 0.39 | 0.50 | 56.89 |
| light chartreuse |  | 0.36 | 0.41 | 75.56 |
| muted chartreuse |  | 0.36 | 0.42 | 41.65 |
| dark chartreuse |  | 0.36 | 0.45 | 19.57 |
| saturated green |  | 0.23 | 0.43 | 29.22 |
| light green |  | 0.28 | 0.36 | 56.92 |
| muted green |  | 0.28 | 0.37 | 29.42 |
| dark green |  | 0.26 | 0.39 | 12.10 |
| saturated cyan |  | 0.22 | 0.33 | 41.79 |
| light cyan |  | 0.26 | 0.33 | 56.95 |
| muted cyan |  | 0.25 | 0.33 | 29.34 |
| dark cyan |  | 0.22 | 0.32 | 12.04 |
| saturated blue |  | 0.20 | 0.23 | 29.02 |
| light blue |  | 0.26 | 0.27 | 56.67 |
| muted blue |  | 0.24 | 0.26 | 29.11 |
| dark blue |  | 0.22 | 0.24 | 12.04 |
| saturated purple |  | 0.28 | 0.14 | 11.63 |
| light purple |  | 0.29 | 0.23 | 41.34 |
| muted purple |  | 0.29 | 0.21 | 19.29 |
| dark purple |  | 0.29 | 0.17 | 6.69 |
